# Synthesis and Cytotoxicity Evaluation of Some Novel Thiazoles, Thiadiazoles, and Pyrido[2,3-*d*][1,2,4]triazolo[4,3-*a*]pyrimidin-5(1*H*)-ones Incorporating Triazole Moiety

**DOI:** 10.3390/molecules20011357

**Published:** 2015-01-14

**Authors:** Sobhi M. Gomha, Sayed A. Ahmed, Abdou O. Abdelhamid

**Affiliations:** 1 Department of Chemistry, Faculty of Science, Cairo University, Giza 12613, Egypt; E-Mail: S.M.Gomha@hotmail.com; 2 Department of Chemistry, Faculty of Science, Beni-Suef University, Beni-Suef 62514, Egypt; E-Mail: skader_70@yahoo.com

**Keywords:** 1,2,3-triazoles, thiazoles, thiadiazoles, pyrido[2,3-*d*][1,2,4]triazolo[4,3-*a*] pyrimidinone, hydrazonoyl halides

## Abstract

Reactions of hydrazonoyl halides and each of methyl 2-(1-(5-methyl-1-phenyl-1*H*-1,2,3-triazol-4-yl)ethylidene)hydrazine-1-carbodithioate and 2-(1-(5-methyl-1-phenyl-1*H*-1,2,3-triazol-4-yl)ethylidene)hydrazine-1-carbothioamide afforded 2-(1-(5-methyl-1-phenyl-1*H*-1,2,3-triazol-4-yl)ethylidene)hydrazono)-3-phenyl-5-substituted-2,3-dihydro-1,3,4-thiadiazoles and 5-(4-substituted)diazenyl)-2-(2-(1-(5-methyl-1-phenyl-1*H*-1,2,3-triazol-4-yl)ethylidene)hydrazinyl)-4-arylthiazoles, respectively. Analogously, the reactions of hydrazonoyl halides with 7-(5-methyl-1-phenyl-1*H*-1,2,3-triazol-4-yl)-5-phenyl-2-thioxo-2,3-dihydropyrido[2,3-*d*]pyrimidin-4(1*H*)-one gave 3-(4-substituted)-8-(5-methyl-1-phenyl-1*H*-1,2,3-triazol-4-yl)-6-phenyl-1-arylpyrido[2,3-*d*]-[1,2,4]-triazolo-[4,3-*a*]pyrimidin-5(1*H*)-ones in a good yield. The structures of the newly synthesized were elucidated via elemental analysis, spectral data and alternative synthesis routes whenever possible. Twelve of the newly synthesized compounds have been evaluated for their antitumor activity against human breast carcinoma (MCF-7) and human hepatocellular carcinoma (HepG2) cell lines. Their structure activity relationships (SAR) were also studied. The 1,3,4-thiadiazole derivative **9b** (IC_50_ = 2.94 µM) has promising antitumor activity against the human hepatocellular carcinoma cell line and the thiazole derivative **12a** has promising inhibitory activity against both the human hepatocellular carcinoma cell line and the breast carcinoma cell line (IC_50_ = 1.19, and 3.4 µM, respectively).

## 1. Introduction

1,2,3-Triazoles are an important class of heterocycles due to their wide range of applications as synthetic intermediates and pharmaceuticals [1,2,3,4]. Several therapeutically interesting 1,2,3-triazoles have been reported, including anti-HIV agents [5,6,7,8], antimicrobial compounds [9], β3-selective adrenergic receptor agonists [10], kinase inhibitors [11,12], other enzyme inhibitors [13,14], the β-lactam antibiotic tazobactam [15] and the cephalosporin cefatrizine [16].

1,3,4-Thiadiazole derivatives have attracted considerable interest owing to their wide spectra of biological activities such as antibacterial, antifungal, antituberculosis, antihepatitis B viral, antileishmanial, anti-inflammatory, analgesic, CNS depressant, anticancer, antioxidant, antidiabetic, molluscicidal, antihypertensive, diuretic, analgesic, antimicrobial, antitubercular, and anticonvulsant activities [17,18,19,20,21,22,23,24,25,26].

Thiazoles can found in drug development for the treatment of allergies [27], hypertension [28], inflammation [29], schizophrenia [30], bacterial [31], HIV infections [32], hypnotics [33] and more recently for the treatment of pain [34], as fibrinogen receptor antagonists with antithrombotic activity [35] and as new inhibitors of bacterial DNA gyrase B [36].

The 1,2,4-triazolopyrimidines have also attracted growing interest due to their important pharmacological activities, such as antitumor potency, antimalarial, antimicrobial, anti-inflammatory, antifungal and macrophage activation [37,38,39,40,41,42]. In continuation of our ongoing work [43,44,45,46,47,48], we report herein the synthesis of some new thiadiazole, thiazole and pyrido[2,3-*d*][1,2,4]triazolo[4,3-*a*]pyrimidine derivatives containing 1,2,3-triazole moieties.

## 2. Results and Discussion

### 2.1. Chemistry

**Scheme 1:**
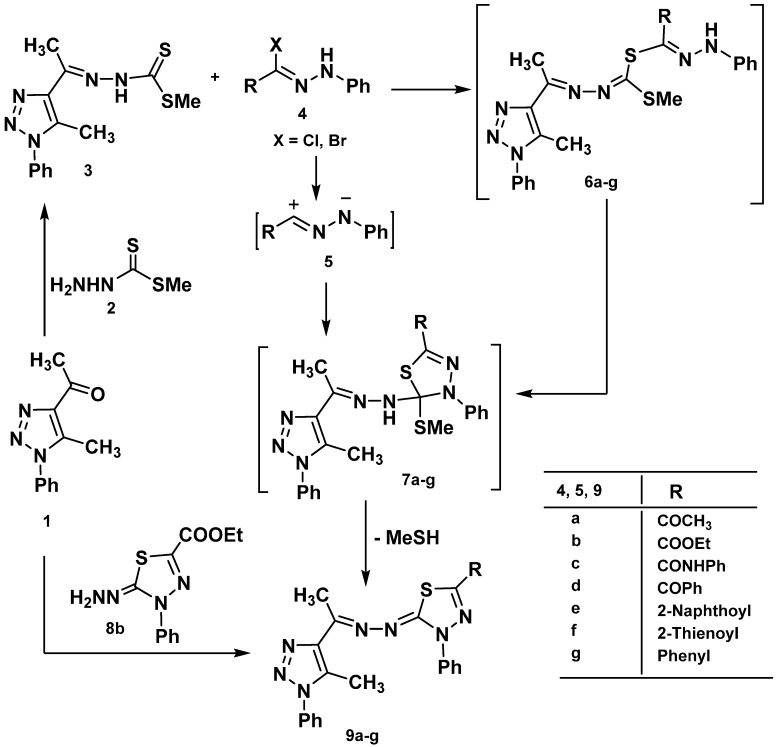
Synthesis of thiadiazoles **9a**–**g**.

Treatment of 4-acetyl-5-methyl-1-phenyl-1*H*-1,2,3-triazole (**1**) [49] with methyl hydrazino-carbodithioate (**2**) in 2-propanol afforded methyl 2-(1-(5-methyl-1-phenyl-1*H*-1,2,3-triazol-4-yl)ethylidene)hydrazinecarbodithioate (**3**) (Scheme 1). Structure **3** was elucidated by elemental analysis, spectral analysis, and chemical transformation. Compound **3** when reacted with ethyl 2-chloro-2-(2-phenylhydrazono)acetate (**4b**) in ethanolic triethylamine at room temperature gave one isolated product formulated as ethyl 5-((1-(5-methyl-1-phenyl-1*H*-1,2,3-triazol-4-yl)-ethylidene)hydrazono)-4-phenyl-4,5-dihydro-1,3,4-thiadiazole-2-carboxylate (**9b**). Structure **9b** was confirmed by elemental analysis, spectral data, and an alternative synthesis route. Thus, 2,3-dihydro-1,3,4-thiadiazole **8b** [50] was reacted with 4-acetyl-5-methyl-1-phenyl-1*H*-1,2,3-triazole (**1**) in ethanol afforded a product identical to **9b** in all aspects (m.p., mixed m.p., and spectra). In the light of the these results, the mechanism outlined in Scheme 1 seems to be the most plausible pathway for the formation of **9b** from the reaction of the **3** with **4b**. The reaction involves initial formation of thiohydrazonate **6**, which undergoes intermolecular cyclization as soon as it is formed to yield the intermediate **7** or via 1,3-dipolar cycloaddition of nitrileimine **5b** [prepared *in situ* from **4b** with triethylamine] to the C=S double bond of **3**. The formation of **6** and **7** are similar to the reactions of hydrazonoyl chloride with 1-phenyl-1,4-dihydrotetrazole-5-thione [51] and 5-phenyl-1,3,4-thiadiazole-2(3*H*)-thione [52]. Compound **7** was converted to **9** by elimination of methanthiol. Analogously, treatment of the appropriate **3** with each of **4a**, **4c**–**g** gave 2,3-dihydro-1,3,4-thiadiazoles **9b**–**g**, respectively, in good yield (Scheme 1).

**Scheme 2:**
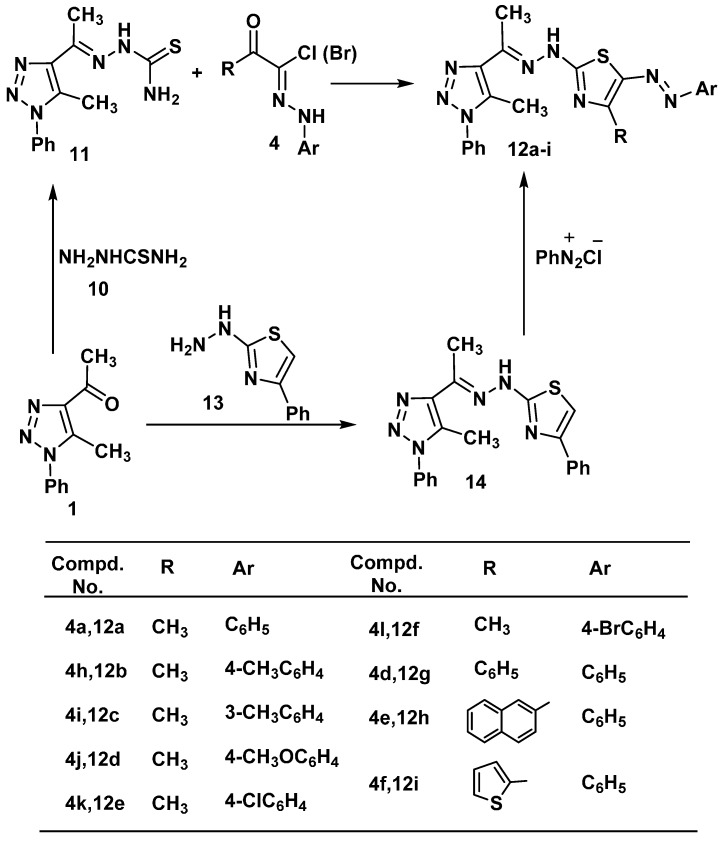
Synthesis of thiazolederivatives **12a**–**i**.

Reaction of 4-acetyl-5-methyl-1-phenyl-1*H*-1,2,3-triazole (**1**) with thiosemicarbazide (**10**) in ethanol afforded 2-(1-(5-methyl-1-phenyl-1*H*-1,2,3-triazol-4-yl)ethylidene)hydrazinecarbothioamide (**11**) in a good yield. The structure of **11** was elucidated via elemental analysis, spectral data and chemical transformation. Its ^1^H-NMR showed signals at δ 2.47 (s, 3H, CH_3_), 2.68 (s, 3H, CH_3_), 6.67 (s, br., 2H, NH_2_), 7.32–7.58 (m, 5H, ArH’s), 8.73 (s, br., 1H, NH). Compound **11** was reacted hydrazonoyl chloride **4a** in ethanol under refluxed gave the corresponding 4-methyl-2-(2-(1-(5-methyl-1-phenyl-1*H*-1,2,3-triazol-4-yl)ethylidene)hydrazinyl)-5-(phenyldiazenyl)thiazole (**12a**) in quantitative yield (Scheme 2). Structure **12a** was confirmed by elemental analysis, spectral data and alternative synthesis. Thus, 2-(2-(1-(5-methyl-1-phenyl-1*H*-1,2,3-triazol-4-yl)ethylidene)hydrazinyl)-4-phenyl-thiazole (**14**), which was prepared from reaction of **1** with 2-hydrazinyl-4-phenylthiazole (**13**) [53], was coupled with benzenediazonium chloride in ethanolic sodium acetate at 0–5 °C to afford a product identical in all respects (mp, mixed mp, and spectra) to **12a**. Analogously, treatment of **11** with the appropriate **4** gave thiazole derivatives**12b**–**i**, respectively, in good yield (Scheme 2).

**Scheme 3:**
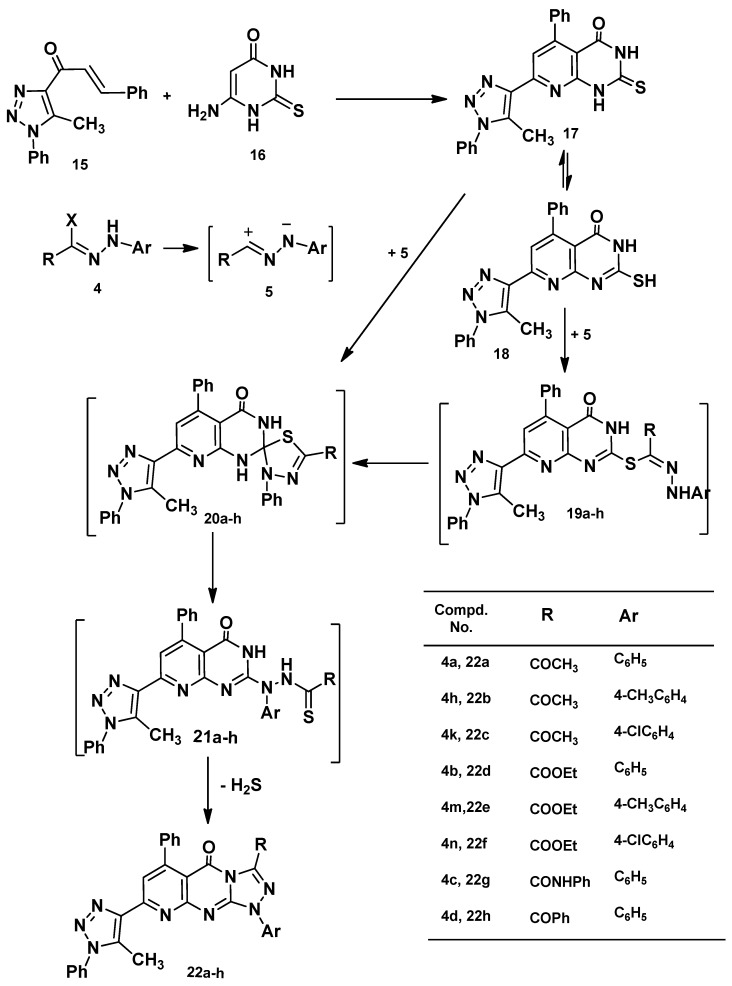
Synthesis of pyrido[2,3-*d*][1,2,4]triazolo[4,3-*a*]pyrimidin-5(1*H*)-ones **22a**–**h**.

Next, 1-(5-methyl-1-phenyl-1*H*-1,2,3-triazol-4-yl)-3-phenylprop-2-en-1-one (**15**) [54] was reacted with 6-amino-2-thioxo-2,3-dihydropyrimidin-4(1*H*)-one (**16**) in ethanol to afford 7-(5-methyl-1-phenyl-1*H*-1,2,3-triazol-4-yl)-5-phenyl-2-thioxo-2,3-dihydropyrido[2,3-*d*]pyrimidin-4(1*H*)-one (**17**) in a good yield. Structure **17** was elucidated by elemental analysis, spectral data and chemical transformation. Thus, when compound **17** was reacted with **4a** in chloroform under reflux it afforded one isolable product, as evidenced by tlc, formulated as 3-acetyl-8-(5-methyl-1-phenyl-1*H*-1,2,3-triazol-4-yl)-1,6-diphenylpyrido[2,3-*d*][1,2,4]triazolo[4,3-*a*]pyrimidin-5(1*H*)-one (**22a**, Scheme 3). The mechanism outlined in Scheme 3 seems to be the most plausible pathway for the formation of **22** from the reaction of thione **17** with **4** via two pathways: (1) 1,3-addition of the thiol tautomer **18** to the nitrilium imide **5** to give the thiohydrazonate ester **19** which undergoes nucleophilic cyclization to yield spiro compounds **20**. The latter ring opened and cyclized to yield **22** by loss of hydrogen sulfide; and (2) 1,3-cycloaddition of nitrilium imide **5** to the C=S double bond of **17** to give **20** directly (Scheme 3). Attempts to isolate the thiohydrazonate ester **19**, spiro intermediate **20** and thiohydrazide **21** did not succeed, even under mild conditions as they readily undergo *in situ* cyclization followed by elimination of hydrogen sulfide to give the final product **22** in Scheme 3.

### 2.2. Cytotoxic Activity

**Figure 1:**
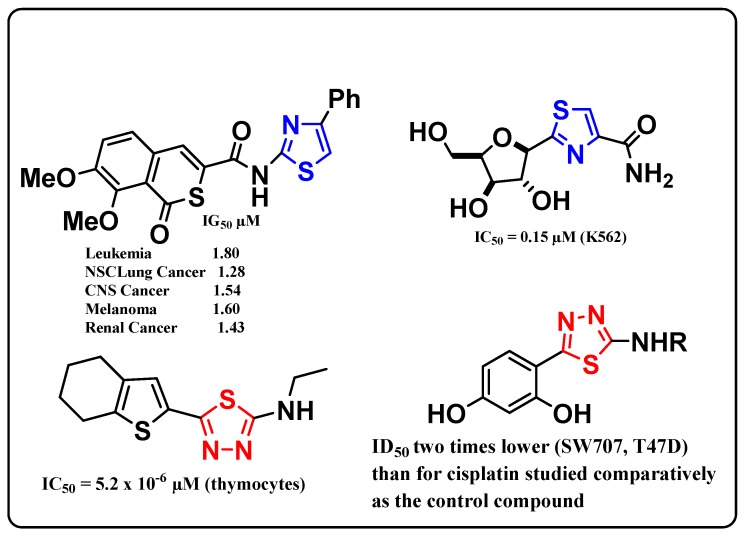
Antitumor activity of thiazoles and 1,3,4-thiadiazoles.

Our literature survey showed that many thiazole and 1,3,4-thiadiazole derivatives have antitumor activity with excellent IG_50_ and IC_50_ values, as depicted in Figure 1 [55,56,57,58]. In view of these facts, we examined the antitumor activity of a new series of substituted thiadiazoles and thiazoles against the human breast carcinoma cell line (MCF-7) and against the human hepatocellular carcinoma (HepG2).

**Table 1:** The *in vitro* inhibitory activity of tested compounds against tumor cell lines expressed as IC_50_ values (μM) ± standard deviation from six replicates.

Tested Compounds	Tumor Cell Lines	
	MCF-7	HepG2
**9a**	29.11 ± 0.21	25.42 ± 0.21
**9b**	8.67 ± 0.30	2.94 ± 0.12
**9c**	7.72 ± 0.18	17.60 ± 0.23
**9d**	22.40 ± 0.20	16.13 ± 0.21
**9e**	22.94 ± 0.18	21.72 ± 0.14
**9f**	38.21 ± 0.16	43.43 ± 0.19
**9g**	19.72 ± 0.20	10.71 ± 0.27
**12a**	3.4 ± 0.23	1.19 ± 0.07
**12b**	22.5 ± 0.24	27.90 ± 0.24
**12c**	20.1 ± 0.12	29.41± 0.07
**22a**	37.7 ± 0.11	27.94 ± 0.13
**22d**	39.9 ± 0.07	43.62 ± 0.14
**Doxorubicin**	0.46 ± 0.21	0.42 ± 0.22

**Figure 2:**
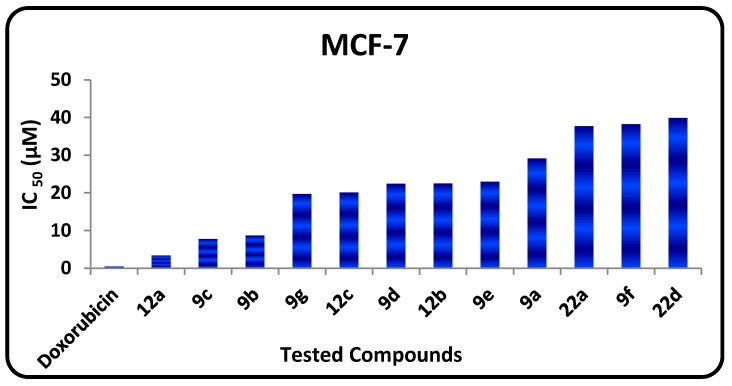
IC_50_ values of tested compounds against MCF-7.

**Figure 3:**
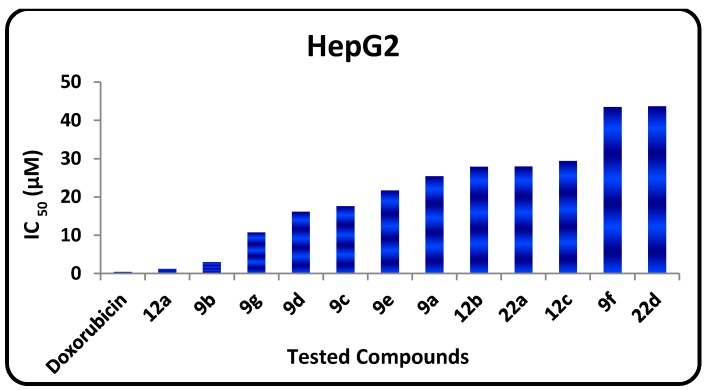
IC_50_ values of tested compounds against HepG2.

The *in vitro* growth inhibitory activity of the synthesized compounds was investigated in comparison with the well-known anticancer standard drug doxorubicin using a crystal violet colorimetric viability assay. Data generated were used to plot a dose response curve of which the concentration of test compounds required to kill 50% of the cell population (IC_50_) was determined. The cytotoxic activity was expressed as the mean IC_50_ of three independent experiments (Table 1) and the results revealed that all the tested compounds showed inhibitory activity to the tumor cell lines in a concentration dependent manner. The small values of IC_50_ for the selected compounds indicate that, for more anticancer effect higher concentrations can be used. The results are represented in Table 1, Figure 2 and Figure 3 show that:

- The *in vitro* inhibitory activities of tested compounds against the human breast carcinoma (MCF-7) have the following descending order: **12a** > **9c** > **9b** > **9g** > **12e** > **9d** > **12b** > **9e** > **9a** > **22d** > **9f** > **22a**.
- The *in vitro* inhibitory activities of tested compounds against the human hepatocellular carcinoma (HepG2) cell line have the following descending order: **12a** > **9b** > **9g** > **9d** > **9c** > **9e** > **9a** > **12e** > **22d** > **12b** > **9f** > **22a**.

Examination of the SAR leads to the following conclusions:

- The 1,3,4-thiadiazole **9b** (IC_50_ = 2.94 µM) has promising antitumor activity against the human hepatocellular carcinoma cell line while the other 1,3,4-thiadiazole derivatives **9a**, **9c**–**f** have moderate activities (IC_50_ = 7.72‒43.43 µM).
- Thiazole **12a** has promising inhibitory activity against both the human hepatocellular carcinoma cell line and the breast carcinoma cell line (IC_50_ = 1.19, and 3.4 µM, respectively) while the other thiazole derivatives **12b** and **12e** have moderate activity.
- Pyridotriazolopyrimidinone derivatives **22a**,**d** have moderate activity.
- For substituents at position 2 of the 1,3,4-thiadiazole ring, the *in vitro* inhibitory activity of tested compounds against the human breast carcinoma cell line have the following descending order: CONHC_6_H_5_ > COOC_2_H_5_ > C_6_H_5_ > C_6_H_5_CO > C_10_H_7_CO > CH_3_CO > C_4_H_3_SCO group.
- For substituents at position 2 of the 1,3,4-thiadiazole ring, the *in vitro* inhibitory activity of tested compounds against the human hepatocellular carcinoma cell line have the following descending order: COOC_2_H_5_ > C_6_H_5_ > C_6_H_5_CO > CONHC_6_H_5_ > C_10_H_7_CO > CH_3_CO > C_4_H_3_SCO group.

## 3. Experimental Section

### 3.1. Chemistry

#### 3.1.1. General

Melting points were measured on an Electrothermal IA 9000 series digital melting point apparatus. IR spectra were recorded in potassium bromide discs on PyeUnicam SP 3300 and Shimadzu FTIR 8101 PC infrared spectrophotometers. NMR spectra were recorded on a Varian Mercury VX-300 NMR spectrometer operating at 300 MHz (^1^H-NMR) and run in deuterated dimethylsulfoxide (DMSO-*d*_6_). Chemical shifts were related to that of the solvent. ^13^C-NMR was recorded on a Bruker spectrometer at 75 MHz. Mass spectra were recorded on a Shimadzu GCMS-QP1000 EX mass spectrometer at 70 eV. Elemental analyses were measured by using an ElementarVario LIII CHNS analyzer. Antitumor activity of the productswas carried out at the Regional Center for Mycology and Biotechnology at Al-Azhar University, Cairo, Egypt. Hydrazonoyl halides **4** [59,60,61,62,63,64,65] were prepared as reported in the respective literature.

#### 3.1.2. Synthesis of Methyl 2-(1-(5-methyl-1-phenyl-1H-1,2,3-triazol-4-yl)ethylidene)hydrazine-1-carbodithioate (**3**)

To a solution of 4-acetyl-5-methyl-1-phenyl-1*H*-1,2,3-triazole (**1**, 2.01 g, l0 mmol) in 2-propanol (20 mL), methyl hydrazinecarbodithioate **2** (1.22 g, 10 mmol) was added. The mixture was stirred at room temperature for 2 h. The solid product was filtered off, recrystallized from ethanol to afford **3** as a yellow solid in 85% yield; mp: 182–184 °C; IR: ν = 3198 (NH), 2993, 2918 (CH), 1601 (C=N) cm^−1^; ^1^H-NMR: δ *=* 2.30 (3H, s, CH_3_), 2.46 (3H, s, CH_3_), 2.67 (3H, s, SCH_3_), 7.56–7.69 (5H, m, Ar-H), 8.38 (1H, s, NH); ^13^C-NMR: δ *=* 14.9 (CH_3_), 17.9 (CH_3_), 21.0 (CH_3_), 116.4, 125.8, 129.6, 129.8, 132.4, 133.1, 134.7, 164.6 (Ar-C), 191.3 (C=S); MS *m/z* (%): 305 (M^+^, 14), 258 (100), 200 (43), 119 (75), 91 (24). Anal. Calcd for C_13_H_15_N_5_S_2_(305.42): C, 51.12; H, 4.95; N, 22.93. Found C, 51.03; H, 4.73; N, 22.74%.

#### 3.1.3. General Procedure for Synthesis of 2-((1-(5-Methyl-1-phenyl-1H-1,2,3-triazol-4-yl)ethylidene)-hydrazono)-3-phenyl-5-subsitituted-2,3-dihydro-1,3,4-thiadiazoles **9a**–**g**

To a mixture of alkyl carbodithioate **3** (0.305 g, 1 mmol) and the appropriate hydrazonoyl halide **4a**–**g** (1 mmol) in ethanol (20 mL), triethylamine (0.5 mL) was added, the mixture was stirred at room temperature for 2 h. The resulting solid was collected and recrystallized from dimethylformamide to give the corresponding 1,3,4-thiadiazolines **9a**–**g**. The products **9a**–**g** together with their physical constants are listed below.

*1-(5-(1-(5-Methyl-1-phenyl-1H-1,2,3-triazol-4-yl)ethylidene)hydrazono)-4-phenyl-4,5-dihydro-1,3,4-thiadiazol-2-yl)ethanone* (**9a**). Yellow solid, (77% yield); mp: 271–273 °C; IR: ν = 3062, 2921 (CH), 1676 (C=O), 1607 (C=N) cm^−1^; ^1^H-NMR: δ = 2.49 (3H, s, CH_3_), 2.50 (3H, s, CH_3_), 2.58 (3H, s, CH_3_), 7.39–8.09 (10H, m, Ar-H); MS, *m/z* (%) 417 (M^+^, 52), 346 (14), 259 (23), 143 (77), 78 (100). Anal. calcd for C_21_H_19_N_7_OS (417.49): C, 60.42; H, 4.59; N, 23.49. Found: C, 60.26; H, 4.51; N, 23.28%.

*Ethyl 5-((1-(5-methyl-1-phenyl-1H-1,2,3-triazol-4-yl)ethylidene)hydrazono)-4-phenyl-4,5-dihydro-1,3,4-thiadiazole-2-carboxylate* (**9b**). Yellow solid, (70% yield); mp: 184–186 °C; IR: ν = 3064, 2983 (CH), 1704 (C=O), 1606 (C=N) cm^−1^; ^1^H-NMR: δ = 1.32 (3H, t, *J* = 7.2, CH_2_CH_3_), 2.49 (3H, s, CH_3_), 2.65 (3H, s, CH_3_), 4.38 (2H, q, *J* = 7.2, CH_2_CH_3_), 7.38–8.01 (10H, m, Ar-H); ^13^C-NMR: δ = 9.6, 11.1, 19.2 CH_3_), 61.2 (CH_2_), 115.2, 116.3, 116.4, 117.2, 118.3, 119.2, 120.6, 120.8, 122.3, 124.4, 126.3, 137.4, 151.2 (Ar-C), 166.3 (CO); MS, *m/z* (%) 447 (M^+^, 17), 346 (6), 289 (11), 170 (49), 143 (69), 118 (27), 78 (100). Anal. calcd for C_22_H_21_N_7_O_2_S (447.51): C, 59.05; H, 4.73; N, 21.91. Found: C, 59.02; H, 4.70; N, 21.79%.

*5-((1-(5-Methyl-1-phenyl-1H-1,2,3-triazol-4-yl)ethylidene)hydrazono)-N,4-diphenyl-4,5-dihydro-1,3,4-thiadiazole-2-carboxamide* (**9c**). Yellow solid, (79% yield); mp: 260–262 °C; IR: ν = 3398 (NH), 3059, 2918 (CH), 1672 (C=O), 1602 (C=N) cm^−1^; ^1^H-NMR: δ = 2.47 (3H, s, CH_3_), 2.65 (3H, s, CH_3_), 7.18–8.08 (15H, m, Ar-H), 11.38 (1H, s, NH); MS, *m/z* (%) 494 (M^+^, 47), 336 (14), 170 (60), 142 (76), 119 (59), 78 (100). Anal. calcd for C_26_H_22_N_8_OS (494.57): C, 63.14; H, 4.48; N, 22.66. Found: C, 63.05; H, 4.40; N, 22.44%.

*5-((1-(5-Methyl-1-phenyl-1H-1,2,3-triazol-4-yl)ethylidene)hydrazono)-4-phenyl-4,5-dihydro-1,3,4-thiadiazol-2-yl)(phenyl)methanone* (**9d**). Yellow solid, (73 yield); mp: 250–252 °C; IR: ν = 3061, 2918 (CH), 1620 (C=O), 1605(C=N) cm^−1^; ^1^H-NMR: δ = 2.43 (3H, s, CH_3_), 2.60 (3H, s, CH_3_), 7.12–7.98 (15H, m, Ar-H);MS, *m/z* (%) 479 (M^+^, 15), 346 (8), 135 (42), 106 (100), 78 (63), 65 (56). Anal. calcd for C_26_H_21_N_7_OS (479.56): C, 65.12; H, 4.41; N, 20.45. Found: C, 65.05; H, 4.37; N, 20.37%.

*(5-((1-(5-Methyl-1-phenyl-1H-1,2,3-triazol-4-yl)ethylidene)hydrazono)-4-phenyl-4,5-dihydro-1,3,4-thiadiazol-2-yl)(naphthalen-2-yl)methanone* (**9e**). Yellow solid, (70% yield); mp: 244–246 °C; IR: ν = 3059, 2928 (CH), 1629 (C=O), 1603 (C=N) cm^−1^; ^1^H-NMR: δ = 2.43 (3H, s, CH_3_), 2.62 (3H, s, CH_3_), 7.13–7.95 (16H, m, Ar-H), 8.22 (s, 1H, naphthalene-H1); MS, *m/z* (%) 529 (M^+^, 31), 512 (100), 324 (64), 155 (57), 135 (58), 78 (89). Anal. calcd for C_30_H_23_N_7_OS (529.61): C, 68.03; H, 4.38; N, 18.51; found: C, 67.89; H, 4.31; N, 18.42%.

*(5-((1-(5-Methyl-1-phenyl-1H-1,2,3-triazol-4-yl)ethylidene)hydrazono)-4-phenyl-4,5-dihydro-1,3,4-thiadiazol-2-yl)(thien-2-yl)methanone* (**9f**). Orange solid, (79% yield); mp: 264–266 °C; IR: ν = 3071, 2920 (CH), 1648 (C=O), 1606 (C=N) cm^−1^;^1^H-NMR: δ = 2.48 (3H, s, CH_3_), 2.62 (3H, s, CH_3_), 7.12–7.97 (13H, m, Ar-H); MS, *m/z* (%) 485 (M^+^, 29), 346 (11), 170 (36), 112 (95), 78 (100). Anal. calcd for C_24_H_19_N_7_OS_2_(485.58): C, 59.36; H, 3.94; N, 20.19. Found: C, 59.28; H, 3.79; N, 20.12%.

*2-((1-(5-Methyl-1-phenyl-1H-1,2,3-triazol-4-yl)ethylidene)hydrazono)-3,5-diphenyl-2,3-dihydro-1,3,4-thiadiazole* (**9g**). Yellow solid, (72% yield); mp: 205–207 °C; IR: ν = 3058, 2916 (CH), 1603 (C=N) cm^−1^; ^1^H-NMR: δ = 2.49 (3H, s, CH_3_), 2.62 (3H, s, CH_3_), 7.10–7.91 (15H, m, Ar-H); ^13^C-NMR: δ *=* 11.4 (CH_3_), 17.3 (CH_3_), 116.0, 116.2, 117.3, 118.2, 118.6, 119.3, 119.6, 120.0, 120.2, 120.6, 122.3, 127.0, 128.5, 128.8, 139.0, 147.7, 151.4 (Ar-C); MS, *m/z* (%) 451 (M^+^, 49), 293 (12), 194 (73), 136 (88), 92(56), 78 (100). Anal. calcd for C_25_H_21_N_7_S (451.55): C, 66.50; H, 4.69; N, 21.71. Found: C, 66.53; H, 4.58; N, 21.64%.

#### 3.1.4. Alternate synthesis of **9b**

To a solution of 4-acetyl-5-methyl-1-phenyl-1*H*-1,2,3-triazole (**1**, 0.201 g, l mmol) in 2-propanol (10 mL), ethyl 5-hydrazono-4-phenyl-4,5-dihydro-1,3,4-thiadiazole-2-carboxylate (**8b**, 0.264 g, 1 mmol) was added. The mixture was refluxed for 2 h then cooled to room temperature. The solid precipitated was filtered off, washed with water, dried and recrystallized from dimethylformamide to give in 69% yield a product which was identical in all aspects (m.p., mixed m.p. and IR spectra) to that obtained from reaction of **3** with **4b**.

#### 3.1.5. Synthesis of 2-(1-(5-Methyl-1-phenyl-1*H*-1,2,3-triazol-4-yl)ethylidene)hydrazinecarbothioamide (**11**)

A mixture of 4-acetyl-5-methyl-1-phenyl-1*H*-1,2,3-triazole (**1**, 2.01 g, 10 mmol) and thio-semicarbazide **10** (0.91 g, 10 mmol) in ethanol (50 mL) containing a catalytic amount of hydrochloric acid was refluxed for 6 h. The desired thiosemicarbazone precipitated from reaction mixture was filtered, washed with ethanol and recrystallized from acetic acid to give pure product of compound **11** as white solid (82%); mp = 221–223 °C; IR: ν *=*3420, 3262, 3191 (NH_2_, NH), 1596 (C=N) cm^−1^; ^1^H-NMR: δ = 2.47 (s, 3H, CH_3_), 2.49 (s, 3H, CH_3_), 3.46 (s, br, 2H, NH_2_), 7.57–7.65 (m, 5H, Ar-H),10.22 (s, br, 1H, NH); MS *m/z* (%): 274 (M^+^, 30), 158 (37), 118 (34), 77 (100). Anal. Calcd: for C_12_H_14_N_6_S (274.34): C, 52.54; H, 5.14; N, 30.63. Found: C, 52.48; H, 5.10; N, 30.48%.

#### 3.1.6. Synthesis of 2-(2-(1-(5-Methyl-1-phenyl-1*H*-1,2,3-triazol-4-yl)ethylidene)hydrazinyl)-5-(aryl-diazenyl)-4-substitutedthiazoles **12a**–**i**

A mixture of thiosemicarbazone **11** (0.274 g, 1 mmol) and the appropriate hydrazonoyl halide **4** (1 mmol) in dioxane (20 mL) containing TEA (0.07 mL) was refluxed for 6 h, allowed to cool and the solid formed was filtered off, washed with EtOH, dried and recrystallized from DMF to give the corresponding 1,3,4-thiadiazolines **12a**–**i**. The products **12a**–**i** together with their physical constants are listed below.

*4-Methyl-2-(2-(1-(5-methyl-1-phenyl-1H-1,2,3-triazol-4-yl)ethylidene)hydrazinyl)-5-(phenyldiazenyl)-thiazole* (**12a**). Red solid, (72% yield); mp 187–189 °C; IR: ν *=* 3414 (NH), 1600 (C=N) cm^−1^; ^1^H-NMR: δ = 2.41 (s, 3H, CH_3_), 2.49 (s, 3H, CH_3_), 2.64 (s, 3H, CH_3_), 7.18–7.92 (m, 11H, Ar-H and NH); ^13^C-NMR: δ *=*10.0, 13.4, 16.5 (CH_3_), 111.6, 117.5, 118.0, 116.2, 122.1, 122.6, 129.0,133.7, 139.3, 144.1, 144.5, 153.6, 154.0, 163.4 (Ar-C); MS, *m/z* (%) 416 (M^+^, 15), 283 (66), 118 (28), 77 (100), 65 (15). Anal. calcd for C_21_H_20_N_8_S (416.50): C, 60.56; H, 4.84; N, 26.90. Found: C, 60.63; H, 4.81; N, 26.76%.

*4-Methyl-2-(2-(1-(5-methyl-1-phenyl-1H-1,2,3-triazol-4-yl)ethylidene)hydrazinyl)-5-(p-tolyldiazenyl)-thiazole* (**12b**). Red solid, (76% yield); mp 193–195 °C; IR: ν *=* 3426 (NH), 1601 (C=N) cm^−1^; ^1^H-NMR: δ = 2.21 (s, 3H, CH_3_), 2.50 (s, 3H, CH_3_), 2.52 (s, 3H, CH_3_), 2.59 (s, 3H, CH_3_), 7.08–7.64 (m, 10H, Ar-H and NH); MS, *m/z* (%)430 (M^+^, 25), 185 (9), 118 (19), 77 (100). Anal. calcd for C_22_H_22_N_8_S (430.53): C, 61.37; H, 5.15; N, 26.03. Found: C, 61.29; H, 5.08; N, 25.84%.

*4-Methyl-2-(2-(1-(5-methyl-1-phenyl-1H-1,2,3-triazol-4-yl)ethylidene)hydrazinyl)-5-(m-tolyldiazenyl)-thiazole* (**12c**). Red solid, (68% yield); mp 156–158 °C; IR: ν *=* 3435 (NH), 1600 (C=N) cm^−1^; ^1^H-NMR: δ 2.20 (s, 3H, CH_3_), 2.46 (s, 3H, CH_3_), 2.52 (s, 3H, CH_3_), 2.57 (s, 3H, CH_3_), 7.10–7.62 (m, 10H, Ar-H and NH); MS, *m/z* (%)430 (M^+^, 22), 158 (37), 118 (13), 77 (100). Anal. calcd for C_22_H_22_N_8_S (430.53): C, 61.37; H, 5.15; N, 26.03. Found: C, 61.42; H, 5.11; N, 25.87%.

*5-((4-Methoxyphenyl)diazenyl)-4-methyl-2-(2-(1-(5-methyl-1-phenyl-1H-1,2,3-triazol-4-yl)ethylidene)-hydrazinyl)thiazole* (**12d**). Dark red solid, (72% yield); mp 178–180 °C; IR: ν *=* 3431 (NH), 1600 (C=N) cm^−1^; ^1^H-NMR: δ = 2.20 (s, 3H, CH_3_), 2.48 (s, 3H, CH_3_), 2.59 (s, 3H, CH_3_), 3.58 (s, 3H, OCH_3_), 7.13–7.74 (m, 10H, ArH’s and NH); MS, *m/z* (%)446 (M^+^, 35), 158 (54), 118 (39), 107 (35), 77 (100). Anal. calcd for C_22_H_22_N_8_OS (446.53): C, 59.18; H, 4.97; N, 25.09. Found: C, 59.11; H, 4.92; N, 25.02%.

*5-((4-Chlorophenyl)diazenyl)-4-methyl-2-(2-(1-(5-methyl-1-phenyl-1H-1,2,3-triazol-4-yl)ethylidene)-hydrazinyl)thiazole* (**12e**). Orange solid, (76% yield); mp 202–204 °C; IR: ν = 3425 (NH), 1597 (C=N) cm^−1^; ^1^H-NMR: δ = 2.23 (s, 3H, CH_3_), 2.47 (s, 3H, CH_3_), 2.60 (s, 3H, CH_3_), 7.19–7.79 (m, 10H, ArH’s and NH); MS, *m/z* (%) 450 (M^+^, 2), 388 (12), 171 (2), 64 (100). Anal. calcd for C_21_H_19_ClN_8_S (450.95): C, 55.93; H, 4.25; N, 24.85. Found: C, 55.92; H, 4.13; N, 24.76%.

*5-((4-Bromophenyl)diazenyl)-4-methyl-2-(2-(1-(5-methyl-1-phenyl-1H-1,2,3-triazol-4-yl)ethylidene)-hydrazinyl)thiazole* (**12f**). Orange solid, (78% yield); mp 214–216 °C; IR: ν *=* 3422 (NH), 1596 (C=N) cm^−1^; ^1^H-NMR: δ = 2.22 (s, 3H, CH_3_), 2.47 (s, 3H, CH_3_), 2.60 (s, 3H, CH_3_), 7.17–7.82 (m, 10H, ArH’s and NH); MS, *m/z* (%) 494 (M^+^, 14), 414 (31), 171 (16), 158 (56), 142 (63), 77 (100). Anal. calcd for: C_21_H_19_BrN_8_S (495.40): C, 50.91; H, 3.87; N, 22.62. Found: C, 50.79; H, 3.77; N, 22.49%.

*2-(2-(1-(5-Methyl-1-phenyl-1H-1,2,3-triazol-4-yl)ethylidene)hydrazinyl)-4-phenyl-5-(phenyldiazenyl)-thiazole* (**12g**). Orange solid, (73% yield); mp 198–200 °C; IR: ν *=* 3429(NH), 1596 (C=N) cm^−1^; ^1^H-NMR: δ = 2.49 (s, 3H, CH_3_), 2.64 (s, 3H, CH_3_), 7.32–8.23 (m, 16H, ArH’s and NH); MS, *m/z* (%) 478 (M^+^, 12), 171 (31), 158 (34), 130 (10), 118 (29), 77 (100). Anal. calcd for C_26_H_22_N_8_S (478.57): C, 65.25; H, 4.63; N, 23.41.Found: C, 65.18; H, 4.60; N, 23.27%.

*2-(2-(1-(5-Methyl-1-phenyl-1H-1,2,3-triazol-4-yl)ethylidene)hydrazinyl)-4-(naphthalen-2-yl)-5-(phenyldiazenyl)thiazole* (**12h**). Red solid, (67% yield); mp 187–189 °C; IR: ν *=* 3439 (NH), 1595 (C=N) cm^−1^; ^1^H-NMR: δ = 2.24 (s, 3H, CH_3_), 2.62 (s, 3H, CH_3_), 7.17–7.83 (m, 17H, ArH’s and NH), 8.12 (s, 1H, naphthalene-H1); MS, *m/z* (%) 528 (M^+^, 2), 484 (6), 286 (8), 244 (4), 127 (39), 77 (100). Anal. calcd for C_30_H_24_N_8_S (528.63): C, 68.16; H, 4.58; N, 21.20.Found: C, 68.11; H, 4.46; N, 21.03%.

*2-(2-(1-(5-Methyl-1-phenyl-1H-1,2,3-triazol-4-yl)ethylidene)hydrazinyl)-5-(phenyldiazenyl)-4-(thien-2-yl)thiazole* (**12i**). Dark red solid, (70% yield); mp 176–178 °C; IR: *υ* ν *=* 3424 (NH), 1599 (C=N) cm^−1^; ^1^H-NMR: δ = 2.23 (s, 3H, CH_3_), 2.60 (s, 3H, CH_3_), 7.10–7.75 (m, 14H, ArH’s and NH); MS, *m/z* (%) 484 (M^+^, 11), 158 (31), 142 (15), 118 (26), 77 (100). Anal. calcd for C_24_H_20_N_8_S_2_(484.60): C, 59.48; H, 4.16; N, 23.12.Found: C, 59.49; H, 4.11; N, 23.03%.

#### 3.1.7. Synthesis of 2-(2-(1-(5-Methyl-1-phenyl-1*H*-1,2,3-triazol-4-yl)ethylidene)hydrazinyl)-4-phenylthiazole (**14**) 

To a solution of 4-acetyl-5-methyl-1-phenyl-1*H*-1,2,3-triazole (**1**, 0.201 g, l mmol) in 2-propanol (10 mL), 2-hydrazinyl-4-phenylthiazole (**13**, 0.191 g, 1 mmol) was added. The mixture was refluxed for 2 h then cooled to room temperature. The solid product was filtered off, washed with ethanol and recrystalized from ethanol to afford the thiazole derivative **14** as a white solid, (73% yield); mp 182–184 °C; IR: *υ =* 3198 (NH), 1603 (C=N) cm^−1^; ^1^H-NMR: δ = 2.47 (s, 3H, CH_3_), 2.60 (s, 3H, CH_3_), 7.24–7.77 (m, 12H, ArH’s, thiazole H-5 and NH); MS, *m/z* (%) 374 (M^+^, 12), 230 (73), 158 (36), 104 (63), 77 (100). Anal. calcd for C_20_H_18_N_6_S (374.46): C, 64.15; H, 4.85; N, 22.44.Found: C, 64.10; H, 4.69; N, 22.31%.

#### 3.1.8. Alternate Synthesis of **12g**

To a solution of **14** (0.374 g, 1 mmol) in ethanol (20 mL) was added sodium acetate trihydrate (0.138 g, 1 mmol), and the mixture was cooled to 0–5 °C in an ice bath. To the resulting cold solution was added portionwise a cold solution of benzenediazonium chloride [prepared by diazotizing aniline (1 mmol) dissolved in hydrochloric acid (6 M, 1 mL) with a solution of sodium nitrite (0.07 g, 1 mmol) in water (2 mL). After complete addition of the diazonium salt, the reaction mixture was stirred for a further 30 min in an ice bath. The solid that separated was filtered off, washed with water and finally recrystallized from DMF to give a 76% of a product which was identical in all aspects (m.p., mixed m.p. and IR spectra) with those obtained from reaction of **11** with **4d**.

#### 3.1.9. Synthesis of 7-(5-Methyl-1-phenyl-1*H*-1,2,3-triazol-4-yl)-5-phenyl-2-thioxo-2,3-dihydropyrido-[2,3-*d*]pyrimidin-4(1*H*)-one (**17**)

A mixture of 1-(5-methyl-1-phenyl-1*H*-1,2,3-triazol-4-yl)-3-phenylprop-2-en-1-one (**15**, 2.89 g, 10 mmol) and 6-amino-2-thioxo-2,3,4-trihydro-1*H*-pyrimidin-4-one (**16**, 1.43 g, 10 mmol) in glacial acetic acid (30 mL) was heated under reflux for 5 h. After cooling, the reaction mixture was poured into ice/HCl mixture and the formed solid was collected and recrystallized from DMF to give thione **17** as yellow crystals, 79%, mp 253–255 °C; IR: ν = 3425, 3205 (2NH), 1668 (C=O), 1598 (C=N) cm^−1^; ^1^H-NMR: δ = 2.41(s, 3H, CH_3_), 7.15–8.24 (m, 11H, ArH’s and pyridine-H), 11.41 (br.s, 1H, NH), 11.93 (s, br., 1H, NH); MS, *m/z* (%) 412 (M^+^, 51), 294 (63), 209 (71), 149 (19), 66 (16); Anal. Calcd. For C_22_H_16_N_6_OS (412.47): C, 64.06; H, 3.91; N, 20.38. Found: C, 64.06; H, 3.91; N, 20.38%.

#### 3.1.10. General Procedure for Synthesis of Pyrido[2,3-*d*][1,2,4]triazolo-[4,3-*a*] pyrimidin-5(1*H*)-ones **22a**–**h**

To a solution of **17** (0.412 g, 1 mmol) and the appropriate hydrazonoyl halides **4** (1 mmol) in dioxane (20 mL) was added triethylamine (0.14 mL, 1 mmol). The reaction mixture was refluxed till all of the starting materials had disappeared (20–24 h, monitored by TLC). The solvent was evaporated and the residue was triturated with methanol. The solid formed was collected and recrystallized from the appropriate solvent to give products **22a**–**h**. The products **22a**–**h** together with their physical constants are listed below.

*3-Acetyl-8-(5-methyl-1-phenyl-1H-1,2,3-triazol-4-yl)-1,6-diphenylpyrido[2,3-d][1,2,4]triazolo-[4,3-a] pyrimidin-5(1H)-one* (**22a**). Yellow solid, (82% yield), mp 262–264 °C; IR: ν = 1670, 1651 (2C=O), 1599 (C=N) cm^−1^; ^1^H-NMR: δ = 2.42 (s, 3H, CH_3_), 2.68 (s, 3H, CH_3_),7.30–8.38 (m, 16H, ArH’s and pyridine-H); MS, *m/z* (%) 538 (M^+^, 17), 373 (18), 260 (23), 156 (21), 80 (100), 56 (23). Anal. Calcd. forC_31_H_22_N_8_O_2_ (538.56): C, 69.13; H, 4.12; N, 20.81. Found: C, 69.08; H, 4.02; N, 20.68%.

*3-Acetyl-8-(5-methyl-1-phenyl-1H-1,2,3-triazol-4-yl)-6-phenyl-1-(p-tolyl)pyrido[2,3-d][1,2,4]triazolo [4,3-a]pyrimidin-5(1H)-one* (**22b**). Yellow solid, (76% yield), mp 262–264 °C; IR: ν = 1721, 1670 (2C=O), 1601(C=N) cm^−1^; ^1^H-NMR: δ = 2.26 (s, 3H, CH_3_), 2.41 (s, 3H, CH_3_), 2.64 (s, 3H, CH_3_), 7.14–7.99 (m, 15H, ArH’s and pyridine-H); MS, *m/z* (%) 552 (M^+^, 23), 515 (23), 370 (25), 217 (28), 106 (79), 52 (100). Anal. Calcd. For C_32_H_24_N_8_O_2_ (552.59): C, 69.55; H, 4.38; N, 20.28. Found: C, 69.38; H, 4.19; N, 20.21%.

*3-Acetyl-1-(4-chlorophenyl)-8-(5-methyl-1-phenyl-1H-1,2,3-triazol-4-yl)-6-phenylpyrido[2,3-d] [1,2,4]triazolo[4,3-a]pyrimidin-5(1H)-one* (**22c**). Yellow solid, (79% yield), mp 278–280 °C; IR: ν = 1721, 1670 (2C=O), 1600 (C=N) cm^−1^; ^1^H-NMR: δ = 2.42 (s, 3H, CH_3_), 2.64 (s, 3H, CH_3_), 7.19–7.97 (m, 15H, ArH’s and pyridine-H); MS, *m/z* (%) 573 (M^+^, 5), 213 (25), 129 (32), 98 (100), 57 (94). Anal. Calcd. For C_31_H_21_ClN_8_O_2_ (573.00): C, 64.98; H, 3.69; N, 19.56. Found: C, 64.75; H, 3.61; N, 19.44%.

*Ethyl 8-(5-methyl-1-phenyl-1H-1,2,3-triazol-4-yl)-5-oxo-1,6-diphenyl-1,5-dihydropyrido[2,3-d] [1,2,4]triazolo[4,3-a]pyrimidine-3-carboxylate* (**22d***)*. Yellow solid, (73% yield), mp 246–248 °C; IR: ν = 1749, 1670 (2C=O), 1601 (C=N) cm^−1^; ^1^H-NMR: δ = 1.29 (t, *J* = 7.2, 3H, CH_3_), 2.40 (s, 3H, CH_3_), 4.13 (q, *J* = 7.2, 2H, CH_2_), 7.17–7.64 (m, 16H, Ar-H and pyridine-H) ppm; MS, *m/z* (%) 568 (M^+^, 14), 481 (19), 236 (17), 111 (32), 69 (10), 55 (100). Anal. Calcd. For C_32_H_24_N_8_O_3_ (568.58): C, 67.60; H, 4.25; N, 19.71. Found: C, 67.43; H, 4.20; N, 19.58%.

*Ethyl 8-(5-methyl-1-phenyl-1H-1,2,3-triazol-4-yl)-5-oxo-6-phenyl-1-(p-tolyl)-1,5-dihydropyrido[2,3-d] [1,2,4]triazolo[4,3-a]pyrimidine-3-carboxylate* (**22e**). Yellow solid, (71% yield), mp 225–227 °C; IR: ν = 1750, 1653 (2C=O), 1601 (C=N) cm^−1^; ^1^H-NMR: δ = 1.32 (t, *J* = 7.2, 3H, CH_3_), 2.24 (s, 3H, CH_3_), 2.41 (s, 3H, CH_3_), 4.16 (q, *J* = 7.2, 2H, CH_2_), 7.12–7.83 (m, 15H, Ar-H and pyridine-H); MS, *m/z* (%) 582 (M^+^, 22), 431 (20), 222 (33), 131 (26), 76 (100). Anal. Calcd. For C_33_H_26_N_8_O_3_ (582.61): C, 68.03; H, 4.50; N, 19.23. Found: C, 68.17; H, 4.37; N, 19.07%.

*Ethyl 1-(4-chlorophenyl)-8-(5-methyl-1-phenyl-1H-1,2,3-triazol-4-yl)-5-oxo-6-phenyl-1,5-dihydro-pyrido[2,3-d][1,2,4]triazolo[4,3-a]pyrimidine-3-carboxylate* (**22f**). Yellow solid, (77% yield), mp 270–272 °C; IR: ν = 1750, 1652 (2C=O), 1601 (C=N) cm^−1^; ^1^H-NMR: δ = 1.34 (t, *J* = 7.2, 3H, CH_3_), 2.43 (s, 3H, CH_3_), 4.21 (q, *J* = 7.2, 2H, CH_2_), 7.18–7.89 (m, 15H, Ar-H and pyridine-H); MS, *m/z* (%) 603 (M^+^, 64), 504 (67), 314 (85), 279 (73), 176 (95), 66 (100). Anal. Calcd. For C_32_H_23_ClN_8_O_3_ (603.03): C, 63.74; H, 3.84; N, 18.58. Found: C, 63.70; H, 3.75; N, 18.47%.

*8-(5-Methyl-1-phenyl-1H-1,2,3-triazol-4-yl)-5-oxo-N,1,6-triphenyl-1,5-dihydropyrido[2,3-d][1,2,4]-triazolo[4,3-a]pyrimidine-3-carboxamide* (**22g**). Yellow solid, (75% yield), mp 270–272 °C; IR: ν = 3325 (NH), 1668, 1651 (2C=O), 1601 (C=N) cm^−1^; ^1^H-NMR: δ = 2.24 (s, 3H, CH_3_), 7.13–8.06 (m, 21H, Ar-H and pyridine-H), 11.27 (br.s, 1H, NH); ^13^C-NMR: δ *=* 10.3 (CH_3_), 120.1, 121.2, 121.3, 121.4, 121.8, 121.9, 125.2, 125.5, 125.8, 126.8, 127.8, 128.2, 128.8, 129.2, 129.6, 129.7, 130.2, 134.1, 135.7, 136.6, 138.0,138.4, 146.6, 148.8, 153.3 (Ar-C), 165.4, 173.6 (C=O); MS, *m/z* (%) 538 (M^+^, 17), 373 (18), 260 (23), 156 (21), 80 (100), 56 (23). Anal. Calcd. For C_36_H_25_N_9_O_2_ (615.64): C, 70.23; H, 4.09; N, 20.48. Found: C, 70.28; H, 4.02; N, 20.27%.

*3-Benzoyl-8-(5-methyl-1-phenyl-1H-1,2,3-triazol-4-yl)-1,6-diphenylpyrido[2,3-d][1,2,4]triazolo[4,3-a]pyrimidin-5(1H)-one* (**22h**). Yellow solid, (78% yield), mp 255–257 °C; IR: ν = 1669, 1652 (2C=O), 1600 (C=N) cm^−1^; ^1^H-NMR: δ = 2.28 (s, 3H, CH_3_), 7.11–8.06 (m, 21H, Ar-H and pyridine-H); MS, *m/z* (%) 600 (M^+^, 27), 504 (32), 300 (40), 148 (87), 95 (59), 67 (100). Anal. Calcd. For C_36_H_24_N_8_O_2_ (600.63): C, 71.99; H, 4.03; N, 18.66. Found: C, 71.69; H, 4.01; N, 18.54%.

### 3.2. Evaluation of the Antitumor Activity Using Viability Assay

Human breast carcinoma (MCF-7) and human hepatocellular carcinoma (HepG2) cell lines were obtained from the American Type Culture Collection (ATCC, Rockville, MD, USA). The cells were grown on RPMI-1640 medium supplemented with 10% inactivated fetal calf serum and 50 µg/mL gentamycin. The cells were maintained at 37 °C in a humidified atmosphere with 5% CO_2_ and were subcultured two to three times a week.

Potential cytotoxicity of the compounds was evaluated on tumor cells using the method of Gangadevi and Muthumary [66]. The cells were grown as monolayers in growth RPMI-1640. The monolayers of 10^4^ cells adhered at the bottom of the wells in a 96-well microtiter plate incubated for 24 h at 37 °C in a humidified incubator with 5% CO_2_. The mono layers were then washed with sterile phosphate buffered saline (0.01 M, pH 7.2) and simultaneously the cells were treated with 100 µL from different dilutions of tested sample in fresh maintenance medium and incubated at 37 °C. A control of untreated cells was made in the absence of tested sample. Positive control containing doxroubcin drug was also tested as reference drug for comparison. Six wells were used for each concentration of the test sample. Every 24 h the observation under the inverted microscope was made. The number of the surviving cells was determined by staining the cells with crystal violet [66,67] followed by cell lysing using 33% glacial acetic acid and reading the absorbance at 590 nm using a microplate reader (SunRise, TECAN, Inc, Männedorf, Switzerland) after mixing well. The absorbance values from untreated cells were considered as 100% proliferation. The number of viable cells was determined using the microplate reader as previously mentioned and the percentage of viability was calculated as [1 − (ODt/ODc)] × 100%, where ODt is the mean optical density of wells treated with the tested sample and ODc is the mean optical density of untreated cells. The relation between surviving cells and drug concentration is plotted to get the survival curve of each tumor cell line after treatment with the specified compound. The 50% inhibitory concentration (IC_50_), the concentration required to cause toxic effects in 50% of intact cells, was estimated from the graphic plots.

## 4. Conclusions

Some newly synthesized compounds were evaluated for their anti-cancer activity against the human breast carcinoma (MCF-7) and human hepatocellular carcinoma (HepG2) cell lines. Also, their structure activity (SAR) was studied. The results revealed that the thiazole derivative **12a** has promising antitumor activities (IC_50_ = 3.41 and 1.12 µM, respectively) and most of the tested compounds showed moderate anti-cancer activities.
